# A nomenclator of Pacific oceanic island *Phyllanthus* (Phyllanthaceae), including Glochidion

**DOI:** 10.3897/phytokeys.4.1581

**Published:** 2011-07-12

**Authors:** Warren L. Wagner, David H. Lorence

**Affiliations:** 1 italic>Department of Botany, MRC-166, National Museum of Natural History, Smithsonian Institution, P.O. Box 37012, Washington, DC 20013-7012; 2 National Tropical Botanical Garden, 3530 Papalina Road, Kalaheo, HI 96741 USA

**Keywords:** Caroline Islands, Fiji, *Glochidion*, Marquesas Islands, Micronesia, Pacific, Phyllanthaceae, *Phyllanthus*

## Abstract

Recent molecular phylogenetic studies and reevaluation of morphological characters have led to the inclusion of *Glochidion* within a broader delimitation of *Phyllanthus*. It is necessary for preparation of the *Vascular Flora of the Marquesas Islands* to make new combinations for the Marquesan species. We also provide the relevant combinations and listing of all of the currently accepted species of *Phyllanthus* on Pacific oceanic islands for a total of 69 native species in oceanic Pacific islands. *Glochidion tooviianum* J. Florenceis here placed into synonymy of *Phyllanthus marchionicus* (F. Br.) W. L. Wagner & Lorence based on new assessment of recently collected specimens from Nuku Hiva. *Glochidion excorticans* Fosberg var. *calvum* Fosberg is placed into synonomy of *Phyllanthus ponapense* (Hosokawa) W. L. Wagner & Lorenceand *Glochidion puberulum* Hosokawa and *Glochidion excorticans* Fosberg are placed in synonymy of *Phyllanthus senyavinianus* (Glassman)W. L. Wagner & Lorence based on new study of all Micronesian specimens available to us. No infraspecific taxa are recognized within *Phyllanthus pacificus* of the Marquesas Islands. Species already with valid names in *Phyllanthus* are also listed for completeness and convenience. Brief distributional comments are given for each species. We propose new names for species for which a new combination is not possible: *Phyllanthus florencei* W. L. Wagner & Lorence, **nom. nov.**, *Phyllanthus mariannensis* W.L. Wagner & Lorence, **nom. nov.**, *Phyllanthus otobedii* W. L. Wagner & Lorence, *Phyllanthus raiateaensis* W. L. Wagner & Lorence, *Phyllanthus st-johnii* W. L. Wagner & Lorence, **nom. nov.,** and *Phyllanthus vitilevuensis* W.L. Wagner & Lorence, **nom. nov.** We provide information for four additional naturalized species within the region (*Phyllanthus amarus*, *Phyllanthus debilis*, *Phyllanthus tenellus*, and *Phyllanthus urinaria*). The name *Glochidion ramiflorum* widely applied to Pacific island populations is here considered to be a species further west in the Pacific with all of the oceanic species here referred to several regional species.

## Introduction

Recent molecular phylogenetic studies have greatly advanced the understanding of relationships in the family Phyllanthaceae (a segregate from Euphorbiaceae *s. l.*) based on chloroplast and nuclear DNA sequence data (Wurdack et al. 2004; Samuel et al. 2005; Kathriarachchi et al. 2005, 2006). Among the tribes in Phyllanthaceae (Hoffmann et al. 2006), tribe Phyllantheae is the largest natural group and accounts for more than half of the 2000 species in the family (Kathriarachchi et al. 2006; Hoffmann et al. 2006). One of the most taxonomically difficult groups in the family is *Phyllanthus* L.and related genera, species of which have small unisexual flowers and an often confusingly similar habit in unrelated groups. Historically generic circumscriptions in Phyllantheae have undergone substantial fluctuation, and the definition of natural groups is still unclear in many parts of the tribe (Hoffmann et al., 2006). The recent molecular studies of relationships within the family, with special emphasis on the large genus *Phyllanthus*, confirm paraphyly of *Phyllanthus* in its traditional circumscription with *Breynia* J. R. Forst. & G. Forst.*, Glochidion* J. R. Forst. & G. Forst.*, Reverchonia* A. Gray, and *Sauropus* Blumeembedded within *Phyllanthus*. These results led the research groups working on Phyllanthaceae (Hoffmann et al. 2006; Kathriarachchi et al. 2006) to conclude that the embedded genera should be submerged into a broadened *Phyllanthus* rather than further generic segregation, which would create a series of highly technical genera distinguishable only by specialists. Distinguishing morphological characters among these five taxa are not clear-cut or comparable, and thus recognition of equivalent clades at generic level would exacerbate the problem of understanding the evolution of this large and widespread lineage (Hoffmann et al. 2006). The enlarged *Phyllanthus* comprises nearly 1300 species with the inclusion of *Breynia*, *Glochidion*, *Reverchonia*, *Phyllanthodendron* Hemsl., and *Sauropus*. It is not surprising that the vast morphological diversity of *Phyllanthus* prior to adding the embedded taxa would readily accommodate them (Hoffmann et al. 2006). Unfortunately, the broadening of *Phyllanthus* necessitates a considerable number of new combinations, but nevertheless this seems to be the best approach over the long term in the interest of developing a classification that facilitates understanding of the evolution and relationships in the overall group.

Our work has focused on the oceanic Pacific and specifically we are currently completing investigation of the flora of the Marquesas Islands as part of a collaboration between National Tropical Botanical Garden (NTBG), Smithsonian Institution (SI), and the Délégation à la Recherche, Papeete, Tahiti (French Polynesia). We do not believe it is wise to make only the necessary combinations for just the Marquesan species previously placed in *Glochidion* so we here provide a synopsis of all of the Pacific oceanic island species (Fiji and Caroline Islands eastward across the Pacific). We also include all of the species currently recognized within *Phyllanthus* for completeness. We have not made an exhaustive study of the taxonomy of these species, but accept for the most part previous taxonomic conclusions. The Pacific group of species is in serious need of comprehensive analysis as previous work has focused on localized areas with few exceptions. Smith (1981) and Florence (1997) have made good regional taxonomic studies, but both point out problems that need a broader geographical perspective to solve. The Micronesian species are in particular need of comprehensive study and have received only partial treatments, either of part of the region (Hosokawa 1935) or more superficial descriptive work (Fosberg and Oliver 1991) without consideration of all species previously described. We have made some changes to the taxonomy of these species, but an in depth study is required to gain a more solid understanding of the diversity within Micronesia. Webster (1986) also made a partial study in the region, primarily of southwestern Pacific species, and Croizat (1943) contributed a useful analysis of several species. We have made extensive use of the Euphorbiaceae world checklist (Govaerts et al. 2000) in compiling the list presented here.

## Systematics

**1.** *Phyllanthus amentuliger* **Müll. Arg.**, Flora 48: 390. 1865. **TYPE. FIJI ISLANDS:** **Vanua Levu:** Mbua Bay, 1840, U.S. Expl. Exped. s.n. (holotype: probably G; Isotype: US!).

*Diasperus amentuliger* (Müll. Arg.) Kuntze, Revis. Gen. Pl. 2: 598. 1891. *Glochidion amentuligerum* Müll. Arg.) Croizat, Sargentia 1: 46. 1942.

**Distribution.** Endemic to Fiji and known from Vanua Levu and eastern Viti Levu at elevations of 100–400 m in dense or open forest, edges, and thickets (Smith 1981).

**2.** *Phyllanthus amicorum* **G. Webster**, Pacific Sci. 40: 100. 1986 [1988]. **TYPE. TONGA:** Eua, E. Soakai 341 (Holotype: K).

**Distribution.** Endemic to the island of Eua, Tonga (Webster 1986) where it is only known from forest margins and exposed rocks, 300m, on the Liku Plateau.

**3.** *Phyllanthus anfractuosus* **(Gibbs) W. L. Wagner & Lorence, comb. nov.**

urn:lsid:ipni.org:names:77112692-1

**Basionym:** *Glochidion anfractuosum* Gibbs, J. Linn. Soc. Bot. 39: 168. 1909. TYPE. FIJI ISLANDS: Viti Levu: Nandarivatu, Sep 1907, *J. G. Gibbs 730* (holotype: BM).

**Distribution.** Endemic to Fiji and known only from Viti Levu and Ovalau at elevations from 100 to 1075 m, in dense or dry forest, thickets or ridge forest (Smith 1981).

**4.** *Phyllanthus aoraiensis* **Nadeaud**, Énum. Pl. Tahiti 73. 1873. **TYPE. SOCIETY ISLANDS: Tahiti:** 1000 m, Nov 1857, J. Nadeaud 459 (holotype: P; isotypes: G [2], P[2]).

**Distribution.** Endemic to the Society Islands and known only from Tahiti at about 1000 m elevation on a ridge crest. Not collected since 1857 and presumed extinct (Florence 1997).

**5.** *Phyllanthus atalotrichus* (**A.C. Sm.) W.L. Wagner & Lorence, comb. nov.**

urn:lsid:ipni.org:names:77112693-1

**Basionym:** *Glochidion atalotrichum* A.C. Sm., Contr. U.S. Natl. Herb. 37: 74. 1967. **TYPE.** **FIJI ISLANDS: Viti Levu:** Namosi, northern Korombasambasanga Range, drainage of Wainavindrau Creek, 450 to 600 m, 28 Sep 1953, A.C. Smith 8747 (holotype: US-02191397!; isotypes: GH, BISH!, L, NY!, S).

**Distribution.** Endemic to Fiji and known only from Namosi Prov., Viti Levu from 250–800 m in dense forest (Smith 1981).

**6.** *Phyllanthus atrovirens* **(A.C. Sm.) W.L. Wagner & Lorence, comb. nov.**

urn:lsid:ipni.org:names:77112694-1

**Basionym:** *Glochidion atrovirens* A.C. Sm., Fl. Vitiensis Nova 2: 481,491. 1981. **TYPE. FIJI ISLANDS: Viti Levu:** Serua, hills between Waininggere and Waisese Creeks, between Ngaloa and Wainiyambia, 50 to 100 m, 10 Dec 1953, A.C. Smith 9550 (holotype: BISH-508142!; isotype: US!).

**Distribution.** Endemic to Fiji and know only from coastal and slightly inland areas of southern Viti Levu from 50–100 m in dry or dense forest (Smith 1981).

**7.** *Phyllanthus bracteatus* **(Gillespie) W.L. Wagner & Lorence, comb. nov.**

urn:lsid:ipni.org:names:77112695-1

**Basionym:** *Glochidion bracteatum* Gillespie, Bernice P. Bishop Mus. Bull. 91: 15. 1932. **TYPE. FIJI ISLANDS: Viti Levu:** Rewa, SE slopes of Mt. Korombamba, 7 Aug 1927, J. W. Gillespie 2169 (holotype: BISH-508144!; isotypes: BISH [2]!).

**Distribution.** Endemic to Fiji from southern and eastern Vitu Levu, from 100 to 430 m in dense or open forest (Smith 1981).

**8.** *Phyllanthus brothersonii* **(J. Florence) W.L. Wagner & Lorence, comb. nov.**

urn:lsid:ipni.org:names:77112696-1

**Basionym:** *Glochidion brothersonii* J. Florence, Fl. Polynésie Française 1: 68. 1997. **TYPE.** **SOCIETY ISLANDS: Raiatea:** Opoa, Mont Oropiro, épaulement nord. 200 m, 151°24’ W, 16°51’ S, 2 Jun 1990, J. Florence 10373 (holotype: P; isotypes: BISH!, CHR, DAV, K, L, P, PAP, PTBG!, US!).

**Distribution.** Endemic to the Society Islands and know only from Raiatea at apparently only low elevations from 200 to 250 m, collected in riparian forest with *Hibiscus tiliaceus* L. and in mesic ridge forest with *Metrosideros collina* (J. R. Forst. & G. Forst.) A. Gray and *Commersonia bartramia* (L.) Merr. (Florence 1997) The area has been converted to pine plantation (D. Hembry, pers. comm. 2011).

**9.** *Phyllanthus brunnescens* **(A.C. Sm.) W.L. Wagner & Lorence, comb. nov.**

urn:lsid:ipni.org:names:77112697-1

**Basionym:** *Glochidion brunnescens* A.C. Sm., Fl. Vitiensis Nova 2: 482, 491. 1981. **TYPE. FIJI ISLANDS: Viti Levu:** Namosi, Mt., Nambui, third peak of Korombasambasanga Range, 12 Nov 1965, DA 14548 pro parte (coll. D. Koroiveibau & I. Qoro) (holotype: BISH-508147!; isotype: SUVA).

**Distribution.** Endemic to Fiji and know only from inland areas of Viti Levu and Vanua Levu, from 300 to 960 m in dense forest, ridge forest and scrubby forest (Smith 1981).

**10.** *Phyllanthus calciphilus* (**Croizat) W.L. Wagner & Lorence, comb. nov.**

urn:lsid:ipni.org:names:77112698-1

**Basionym:** *Glochidion calciphilum* Croizat, Sargentia 1: 46. 1942. **TYPE. FIJI ISLANDS: Fulaga:** limestone cliff of lagoon, 0 to 80 m, 26 Feb 1934, A.C. Smith 1217 (holotype: GH; isotypes: BISH!, K, S, US!).

**Distribution.** Endemic to Fiji and know only from the two islands of southern Lau (Fulaga and Kabara) near sea level on limestone and lagoon cliffs (Smith 1981).

**11.** *Phyllanthus christophersenii* **(Croizat) W. L. Wagner & Lorence, comb. nov.**

urn:lsid:ipni.org:names:77112699-1

**Basionym:** *Glochidion christophersenii* Croizat, Occas. Pap. Bernice P. Bishop Mus. 17(16): 213. 1943. **TYPE. SAMOAN ISLANDS: Savai`i:** above Matavanu, 1300 m, 24 Jul 1931, E. Christophersen & E. P. Hume 2134 (holotype: A; isotype: BISH).

**Distribution.** Endemic to montane Savai`i and known from cloud forest at 1000–1550 m (Whistler 1978; Christophersen 1935).

**12.** *Phyllanthus cleistanthoides* **(Fosberg) W. L. Wagner & Lorence, comb. nov.**

urn:lsid:ipni.org:names:77112700-1

**Basionym:** *Glochidion cleistanthoides* Fosberg, Willdenowia 20: 263. 1991. **TYPE. CAROLINE ISLANDS: Pohnpei:** 1913, C. L. Ledermann 13599a (holotype: B-bc100249519!)

**Distribution.** Endemic to Pohnpei where it occurs from ca. 12 to 770 m elevation, most commonly in lowland wet forest and agroforest, but occasionally in summit cloud forest.

**Note.** This species is distinctive in having oblong-ovate to narrowly oblong-elliptic leaves and comparatively small flowers in umbel-like fascicles often borne on short, sometimes branched stalks or peduncles to 5 mm long, a densely puberulent pistil with a puberulent columnar style exserted for 1–1.5 mm beyond the calyx lobes, and relatively small, densely puberulent fruits. Pohnpei collections of this species have been identified as *Glochidion ramiflorum* or less commonly *Glochidion puberulum*.

**13.** *Phyllanthus comitus* **(J. Florence) W. L. Wagner & Lorence, comb. nov.**

urn:lsid:ipni.org:names:77112701-1

**Basionym:** *Glochidion comitum* J. Florence, Novon 7:29. 1997. **TYPE. PITCAIRN ISLANDS: Pitcairn:** Sommet Crete Sud-Est, 25°4’ S, 130°7’ W, 300 m, 19 Apr 1991, J. Florence 10740 (holotype: K; isotypes: BISH, BM, DAV, E, L, MO, P, PAP, TER, US!).

**Distribution.** Endemic to Pitcairn where it is known from fewer than 10 collections.

**14.** *Phyllanthus concolor* **(Müll. Arg.) Müll. Arg.,** Flora 48: 374. 1865.

**Basionym:** *Glochidion concolor* Müll. Arg., Linnaea 32: 62. 1863. *Phyllanthus concolor* var. *ellipticus* (Müll. Arg.), Prodromus Systematis Naturalis Regni Vegetabilis 15(2): 290. 1866, nom. illeg. *Diasperus concolor* (Müll. Arg.) Kuntze, Revis. Gen. Pl. 2: 599. 1891. **TYPE. FIJI ISLANDS:** *s.l.*, Nov 1855, W. H. Harvey s.n. (holotype: P; isotypes: BM, K).

*Glochidion ramiflorum* J.R. Forst. & G. Forst. var. *lanceolatum* Müll. Arg. Linnaea 32: 63. 1863. *Phyllanthus ramifloris* (J.R. Forst. & G. Forst.) Müll. Arg. var. *lanceolatus* (Müll. Arg.) Müll. Arg., Flora 48: 374. 1865, nom. illeg. **TYPE. FIJI ISLANDS: Ovalau:** Port Kinnaird, June 1860 and Somosomo, Taveuni, May 1860, B.C. Seemann 415 (lectotype: presumably G, designated by Smith (1981, p. 476); isolectotypes: BM, K).\\

**Distribution.** Widespread in Fiji, Tonga, and possibly Raratonga (Smith 1981), and also from Niue (Sykes 1970) from 0 to 1000 m elevation in dense or open forest, edges and forest-grassland transition, and on open slopes. These collections were previously referred to *Glochidion ramiflorum*, butwere considered by Smith (1981) to be a separate species. He considered *Glochidion ramiflorum* to be a species from New Guinea to New Hebrides.

**15.** *Phyllanthus cordatus* **(Seem. ex Müll. Arg.) Müll. Arg.**, Flora 48: 376. 1865.

**Basionym:** *Glochidion cordatum* Seem. ex Müll. Arg., Linnaea 32: 64. 1863. *Diasperus cordatus* (Seem. ex Müll. Arg.) Kuntze, Revis. Gen. Pl. 2: 599. 1891. **TYPE. FIJI ISLANDS: Viti Levu:** July 1860 and **Ovalau**: Port Kinnaird, June 1860 [the K isotype bears 2 field labels], B.C. Seemann 416 (holotype: presumably G; isotypes: BM, K).

**Distribution.** Endemic to Fiji and know from Viti Levu, Ovalau, and Vanua Levu at 50–900 m, in dry forest, ridge forest, and in pastures and along roadsides (Smith 1981).

**16.** *Phyllanthus cuspidatus* **Müll. Arg.**, Flora 48: 377. 1865.

**Basionym:** *Glochidion cuspidatum* (Müll. Arg.) Pax, Bot. Jahrb. Syst. 25: 645. 1898. *Diasperus cuspidatus* (Müll. Arg.) Kuntze, Revis. Gen. Pl. 2: 599. 1891. **TYPE. SAMOAN ISLANDS:** *s.l.*, U.S. Expl. Exped. s.n. (holotype: G-DC).

**Distribution.**  Endemic to the Samoan Islands (Savai`i, `Upolu), and Tutuila at up to 400–450 m in ridge forest (Whistler 1980).

**17.** *Phyllanthus distichus* **Hook. & Arn.**, Bot. Beechey Voy.: 95. 1832.

**Basionym:** *Diasperus distichus* (Hook. & Arn.) Kuntze, Revis. Gen. Pl. 2: 599. 1891. **TYPE. HAWAIIAN ISLANDS:** **O`ahu:** 1827–1827, G.T. Lay & A. Collie s.n. (probable holotype: K; probable isotype: E).

**Distribution.** *Phyllanthus distichus* is endemic to the Hawaiian islands where it is occasional to locally common in mesic forest, often on steep slopes or ridge tops, sometimes in dry shrubland at 60–950 m on the islands of Kaua`i, O`ahu, Moloka`i, Lana`i, West Maui, and rare on East Maui. A number of additional names have been applied to populations of *Phyllanthus distichus*, but they were placed into synonymy by Wagner et al. (1990, 1999) and are not repeated here.

**18.** *Phyllanthus emarginatus* **(J. W. Moore) W. L. Wagner & Lorence, comb. nov.**

urn:lsid:ipni.org:names:77112702-1

**Basionym:** *Glochidion emarginatum* J. W. Moore, Bernice P. Bishop Mus. Bull. 102: 30. 1933. **TYPE.** **SOCIETY ISLANDS: Raiatea:** Mount Temehani, 470 m, 1 Jan 1927, J.W. Moore 476A (holotype: BISH-508150!; isotype: P [2]).

*Glochidion raiateense* J. W. Moore, Bernice P. Bishop Mus. Bull. 102: 30. 1933. **TYPE:** **SOCIETY ISLANDS: Raiatea:** Mount Temehani, 470 m, 1 Jan 1927, J.W. Moore 476B (Holotype: BISH-508230!).

**Distribution.** Endemic to the Society Islands and known only from Raiatea at 580–750 m where it occurs in marshy shrubland with *Metrosideros collina* and species of Cyperaceae. Also collected at ca. 930 m in mesic ridge shrubland with *Ilex* and *Weinmannia* (Florence 1997).

**19.** *Phyllanthus euryoides* **(A. C. Sm.) W. L. Wagner & Lorence, comb. nov.**

urn:lsid:ipni.org:names:77112703-1

**Basionym:** *Glochidion euryoides* A.C. Sm., J. Arnold Arbor. 33: 373. 1952. **TYPE. FIJI ISLANDS: Viti Levu:** Mba, upper slopes of Mt. Koromba (Pickering Peak), 3 Jun 1947, A. C. Smith 4659 (holotype: A; isotypes: BISH!, K, S, US!).

**Distribution.** Endemic to Fiji and known only from the type collection.

**20.** *Phyllanthus florencei* **W. L. Wagner & Lorence, nom. nov.**

urn:lsid:ipni.org:names:77112704-1

**Replaced name:** *Glochidion societatis* J. Florence, Fl. Polynésie Française 1: 90. 1997. **TYPE. SOCIETY ISLANDS: Tahaa:** Patio, Mt. Purauti, crête sud-est, 151°30’ W, 16°37’ S, 225 m, 18 Jun 1990, J. Florence 10,627(holotype: P; isotypes: BISH!, CHR, DAV, K, L, NY!, P [2], PAP, PTBG!, US!).

**Distribution.** Known from the Society Islands (Huahine, Mauapiti, Raiatea, and Tahaa) and Austral Islands (Rimatara) at 0–225 m elevation where it is locally common to abundant in coastal vegetation with *Scaevola* and *Euphorbia* on coral sand, in lowland mesic forest with *Neolauclea* and *Hibiscus tiliaceus*, in mesic ridge forest with *Metrosideros collina* and *Dicranopteris linearis* (Burm. f.) Underw., and in secondary forest (Florence 1997).

**21.** *Phyllanthus gillespiei* **(Croizat) W.L. Wagner & Lorence, comb. nov.**

urn:lsid:ipni.org:names:77112705-1

**Basionym:** *Glochidion gillespiei* Croizat, Sargentia 1: 46. 1942. **TYPE. FIJI ISLANDS:** **Viti Levu:** Namosi, near summit of Mt. Naitarandamu, 28 Sep 1927, J.W. Gillespie 3161 (holotype: GH; isotypes: BISH).

**Distribution.** Endemic to Fiji and known only from mountainous areas of Viti Levu from 750–1155 m in dense or ridge forest (Smith 1981).

**22**. *Phyllanthus grantii* **(J. Florence) W. L. Wagner & Lorence, comb. nov.**

urn:lsid:ipni.org:names:77112706-1

**Basionym:** *Glochidion grantii* J. Florence, Bull. Mus. Natl. Hist. Nat., B, Adansonia 18: 250. 1996. **TYPE. SOCIETY ISLANDS: Tahaa:** Tapuamu, crête entre les Monts Tete et Ohiri. 530 m, 151°31’ W, 16°37’ S, 5 Nov 1992, J. Florence & R. Tahuaitu 11816 (holotype: P; isotype: BISH!, DAV, K, L, PAP, US!).

**Distribution.** Endemic to the Society Islands (Raiatea and Tahaa) where it occurs at 435–730 m elevation in marshy shrubland with *Metrosideros* and Cyperaceae and in wet summit forest with *Metrosideros* and *Macaranga* (Florence 1997).

**23.** *Phyllanthus grayanus* **Müll. Arg.,** Flora 48(24): 380. 1863.

*Diasperus grayanus* (Müll. Arg.) Kuntze, Revis. Gen. Pl. 2: 599 .1891. *Glochidion grayanum* (Müll. Arg.) J. Florence, Bull. Mus. Natl. Hist., B, Adansonia 18: 250. 1996. **TYPE. SOCIETY ISLANDS: Tahiti:** *s. l.*, Sep 1839 or Jan1841, U. S. Expl. Exped. s.n. (Holotype: G-DC).

**Distribution.** Endemic to the Society Islands and known only from Tahiti at 60–1040 m. It occurs in lowland riparian forest with *Neonauclea* and *Hibiscus tiliaceus* and extends up to mesic ridge and summit forest dominated by *Metrosideros collina* (Florence 1997).

**24.** *Phyllanthus heterodoxus* **Müll. Arg.**, in DC., Prodr. 15(2):321. 1866.

*Diasperus heterodoxus* (Müll. Arg.) Kuntze, Revis. Gen. Pl. 2: 599. 1891. *Glochidion heterodoxum* (Müll. Arg.) Pax & K.Hoffm. in H.G.A. Engler, Nat. Pflanzenfam. ed. 2, 19c: 58. 1931. **TYPE. FIJI ISLANDS:** *s. l.*,1840, U.S. Expl. Exped. s.n. (Holotype: Probably G).

**Distribution.** Endemic to Fiji and known only from Vanua Levu and Lau Group, Fulaga from 0 to 870 m (Smith 1981).

**25.** *Phyllanthus hivaoaense* **(J. Florence) W. L. Wagner & Lorence, comb. nov.**

urn:lsid:ipni.org:names:77112707-1

**Figure 1.**
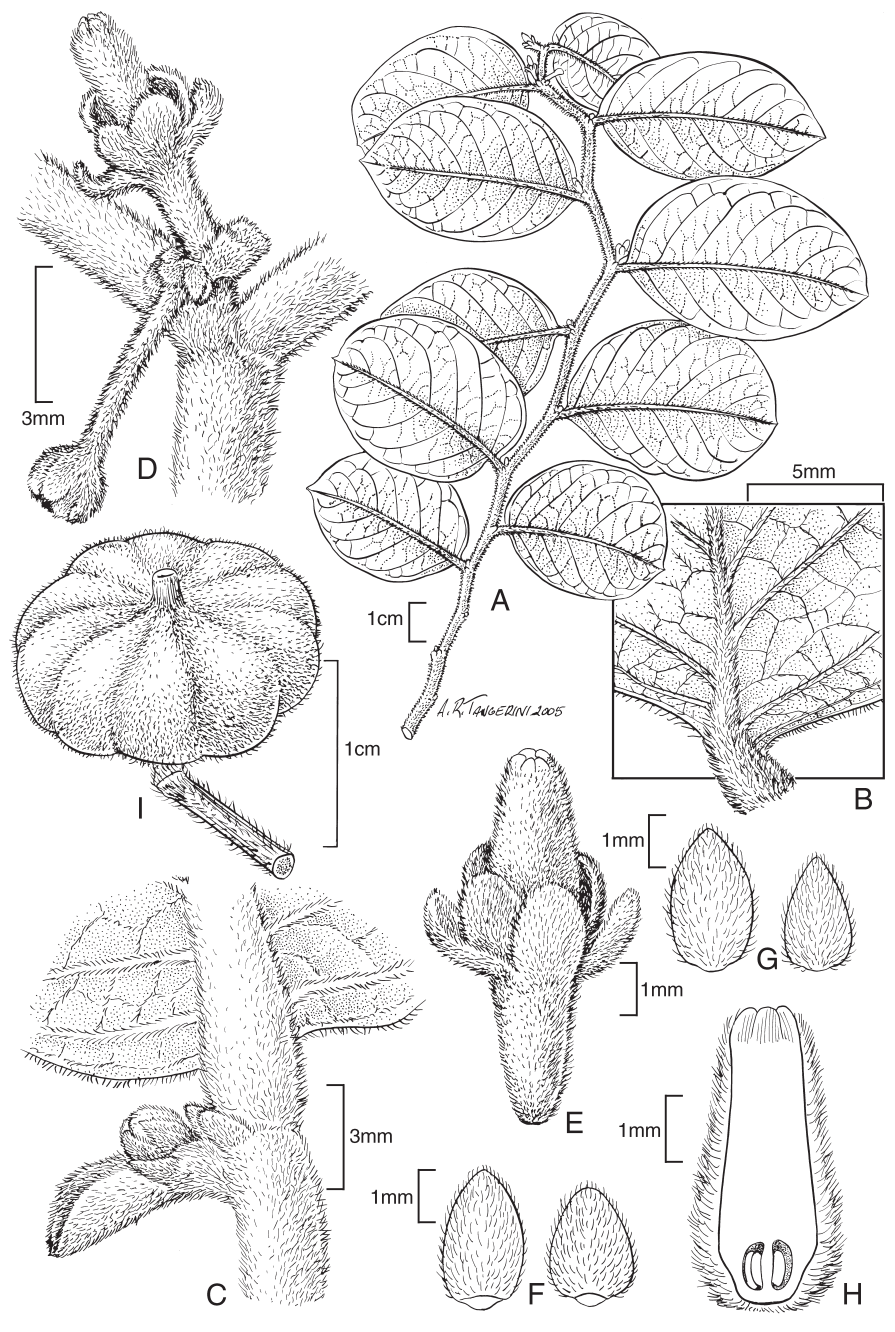
*Phyllanthus hivaoaense* (J. Florence) W. L. Wagner & Lorence. **A** branch **B** abaxial leaf surface **C** leaf axil showing stipules **D** female flowers **E** female flower close-up **F–G** calyx lobes **H** longitudinal view of female flower **I** capsule. **A–I** drawn from: Marquesas Islands. Tahuata: Haaoiputeomo, summit ridge, 823-951 m, Wood et al. 6522(BISH, DAV, K, MO, P, PAP, PTBG, US).

**Basionym:** *Glochidion hivaoaense* J. Florence, Fl. Polynésie Française 1: 74. 1997. **TYPE. MARQUESAS ISLANDS: Hiva Oa:** Atuona, piste de Hanamenu, NW du Mt. Temetiu, 139°5’ W, 9°48’ S, 1100 m, 30 Jul 1988, J. Florence & S. Perlman 9673 (holotype: P; isotypes: BISH!, DAV, K, L, P, PAP, PTBG!, US!). Fig. 1.

**Distribution.** Endemic to the Marquesas Islands of Hiva Oa and Tahuata, from about 700 to 1200 m, collected in wet shrubland and forest with *Freycinetia*, *Weinmannia*, and the tree ferns *Alsophila* and *Sphaeropteris* (Florence 1997).

**26.** *Phyllanthus hosokawae* **(Fosberg) W. L. Wagner & Lorence, comb. nov.**

urn:lsid:ipni.org:names:77112708-1

**Basionym:** *Glochidion hosokawae* Fosberg, Willdenowia 20: 261. 1991. **TYPE: CAROLINE ISLANDS: Pohnpei:** Awak, 25.8.1980, F.R. Fosberg 60467 (holotype: US [not located]: isotypes: BISH, L).

**Distribution.** To date know only from the type collection from Pohnpei; the holotype could not be located at US.

**Note.** This entity should be carefully evaluated in the context of an overall review of *Phyllanthus* in Micronesia. It may be conspecific with *Glochidion cleistanthoides*, in which case we would adopt the latter name because the description applies most closely to this species and the holotype is available at B.

**27.** *Phyllanthus huahineense* **(J. Florence) W. L. Wagner & Lorence, comb. nov.**

urn:lsid:ipni.org:names:77112709-1

**Basionym:** *Glochidion huahineense* J. Florence, Fl. Polynésie Française 1: 75. 1997. **TYPE. SOCIETY ISLANDS: Huahine:** Maeva, motu Oavarei, secteur Haaparu, 151°W, 16°41’ S, 2 m, 1 Nov 1992, J. Florence & R. Tahuaitu 11745 (holotype: P ; isotypes: BISH, DAV, K, L, NY, P, PAP, PTBG !, US !).

**Distribution.** Endemic to the Society Islands and known only from Huahine on the islet Motu de Maeva at 1–4 m elevation, restricted to coral sand substrate in coastal vegetation with *Casuarina*, *Guettarda*, and *Tournefortia* (Florence 1997).

**28.** *Phyllanthus inusitatus* **(A.C. Sm.) W.L. Wagner & Lorence, comb. nov.**

**Basionym:** *Glochidion inusitatum* A.C. Sm., Fl. Vitiensis Nova 2: 486, 493. 1981. **TYPE. FIJI ISLANDS: Vanua Levu:** divide between Wainunu and Ndreketi Rivers, between Nanduna (old village site) and Mt. Ndelanathau, 17 May 1934, A.C. Smith 1851 (holotype: BISH-142932; isotypes: many indicated, but not specific as to where deposited).

**Distribution.** Endemic to Fiji and known only from the type.

**29.** *Phyllanthus jardinii* Müll. Arg., Linnaea 32: 21. 1863. **TYPE.** s. l., D.E.S.A. Jardin s.n. (holotype: G-DC-FP3347; isotypes: P [2]). Type presumed to be from Nuku Hiva in the Marquesas Islands.

urn:lsid:ipni.org:names:77112710-1

**Note.** Precise locality uncertain, known only from a single collection possibly made in the Marquesas Islands (Nuku Hiva) by Jardin in the 19th century (Florence 1997).

**30.** *Phyllanthus kanehirae* **(Hosokawa) W. L. Wagner &** **Lorence, comb. nov.**

urn:lsid:ipni.org:names:77112711-1

**Basionym:** *Glochidion kanehirae* Hosokawa, Trans. Nat. Hist. Soc. Taiwan 25: 22. 1935. **TYPE. CAROLINE ISLANDS: Palau:** Jul–Aug 1929, R. Kanehira 241 (holotype: TAI).

**Distribution.** Caroline Islands: Palau (Peleliu, Koror including the Rock Islands of Mecherchar, Ngeruktabel, Ulebsechel and Urukthapel, and also known from Babeldaob [Fosberg et al. 1979; Wagner et al. unpubl.]), Yap (Yap Island), and Chuuk (Moen Island, also Tol, Udot, Uman, Dublon, Fano, Fanurmot [Fosberg et al. 1979; Wagner et al. unpubl.]). On Palau it is restricted to limestone substrate from near sea level to about 200 m elevation in coastal forest with speices of *Bruguiera*, *Heretiera*, *Semecarpus*, *Osmoxylon*, and *Phyllanthus*, and in lowland evergreen forest with *Horsfieldia* and *Phyllanthus* and agroforest. On Chuuk it occurs near sea level (3 m) in coastal forest interspersed with mangroves and on slopes of unknown elevation in agroforest and secondary vegetation. On Yap it occurs from near sea level at the edge of mangrove vegetation and on slopes up to 40 m with secondary vegetation.

**Note.** This glabrous species is characterized by female flowers with a small, depressed-globose pistil 1–1.5 mm long with a very short 10–11-fid stylar column 0.5 mm long. Certain collections from Yap have been identified as *Glochidion* cf. *ramiflorum*, and certain collections from Palau have been identified as *Glochidion palauense*.

**31.** *Phyllanthus longfieldiae* **L. Riley**, Bull. Misc. Inform. Kew [2]:55. 1926.

*Glochidion longfieldiae* (L. Riley) F. Br., Bernice P. Bishop Mus. Bull. 130: 141. 1935. **TYPE. AUSTRAL ISLANDS: Rapa:** L.A.M. Riley 776 (holotype: K; isotype: BM).

**Distribution.** Endemic to the Austral Islands (Rapa) where it occurs from 140 to 575 m, in mesic *Metrosideros* forest on slopes and in valleys and ravines with species of *Corokia* and *Fitchia* (Florence 1997).

**32.** *Phyllanthus macrosepalus* **(Hosokawa) W. L. Wagner & Lorence, comb. nov.**

urn:lsid:ipni.org:names:77112712-1

**Basionym:** *Glochidion macrosepalum* Hosokawa, Trans. Nat. Hist. Soc. Taiwan 25: 21. 1935. **TYPE. CAROLINE ISLANDS: Palau:** Babeldaob, valley near Mt. Agade, “Arukoron-sogan”, 20 Sep 1933, T. Hosokawa 7053 (holotype: TAI-118940; isotypes: BISH, GH, TAI). Holotype presumed to be the TAI sheet with the typewritten label as is typical of Hosokawa collections.

**Distribution.** Endemic to Palau (Babeldaob, Anguar, Peliliu, and Malakal Islands), where it occurs at 3–150 m, in lowland evergreen moist and wet forest on limestone and limestone-derived soils.

**Note.** This glabrous species is characterized by female flowers with relatively large calyx lobes enclosing the short, depressed-ovoid pistil 1.5 mm long with a very short style column and flat 5-6-sulcate or -lobed stigmatic region. In male flowers the staminal column is composed of 5 connate stamens (versus 3-4 in other Micronesian species).

**33.** *Phyllanthus manono* **(Baill. ex Müll. Arg.) Müll. Arg.**, Flora 48: 377. 1865.

**Basionym:** *Glochidion manono* Baill. ex Müll. Arg.,Linnaea 32: 65. 1863. *Diasperus manono* (Baill. ex Müll. Arg.) Kuntze, Revis. Gen. Pl. 2: 600. 1891. **TYPE. SOCIETY ISLANDS: Tahiti:** *s. l.*,J. Lepine 210 (lectotype: G-DC, designated by Florence, Fl. Polynésie Française 1: 79. 1997; isolectotypes: P [2]).

**Distribution.** Endemic to the Society Islands of Moorea and Tahiti where it occurs at 30–1000 m, in valleys in wet forest with *Neonauclea*, *Hibiscus*, and *Inocarpus* and also on slopes and summits, sometimes in disturbed or secondary forest with *Metrosideros*, *Dicranopteris*, and *Psidium* (Florence 1997).

**34.** *Phyllanthus marchionicus* **(F. Br.) W. L. Wagner & Lorence, comb. nov.**

urn:lsid:ipni.org:names:77112713-1

**Figure 2.**
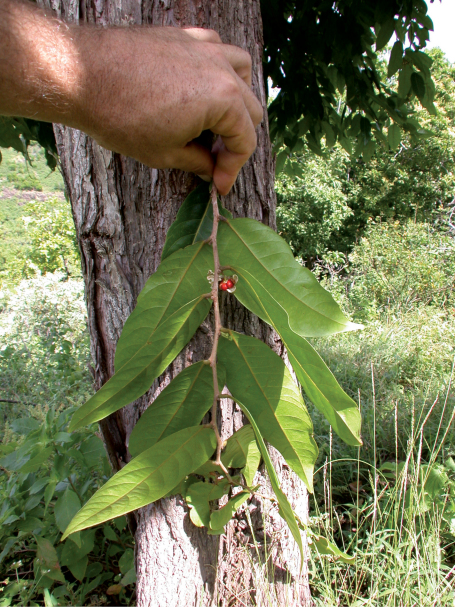
*Phyllanthus marchionicus* (F. Br.) W. L. Wagner & Lorence**.** Branch with dehisced fruit showing red arillate seeds, with trunk in background. Field image by K. R. Wood: Marquesas Islands. Ua Huka, Haahue, northwestern coastal valley,Wood 10765 (PTBG, US).

**Basionym:** *Glochidion marchionicum* F. Br., Bernice P. Bishop Mus. 130: 142. 1935. **TYPE. MARQUESAS ISLANDS: Ua Huka:** 9 Nov 1922, *E. H. Quayle 1689* (Lectotype: BISH-508228! & 508229!, designated by St. John, Phytologia 33: 420. 1976). Fig. 2.

*Glochidion tooviianum* J. Florence, Bull. Mus. Natl. Hist. Nat., B, Adansonia 18: 260. 1996, syn. nov. **TYPE. MARQUESAS ISLANDS: Nuku Hiva:** Toovii, 800 m, 140°9’ W, 8°51’ S, 3 Mar 1986, J. Florence 7445 (holotype: P; isotypes: BISH!, P, PAP, US).

**Distribution.** Endemic to the Marquesas Islands (Nuku Hiva, Ua Huka, Ua Pou, Hiva Oa, Tahuata, and Fatu Hiva) from 50 to 1130 m, where it is widespread in moist valleys with *Hibiscus tiliaceus*, disturbed mesic ridge forest with *Casuarina*, *Sapinus*,and *Xylosma*, and secondary vegetation with *Dicranopteris*, *Leucaena*, *Miscanthus*,and *Psidium* or in primary wet forest with *Cheirodendron*, *Crossostylis*, *Ilex*, and tree ferns (Florence 1997).

**35.** *Phyllanthus mariannensis* **W.L. Wagner & Lorence, nom. nov.**

urn:lsid:ipni.org:names:77112714-1

**Replaced name:** *Glochidion marianum* Müll. Arg., Linnaea 32: 65. 1863. *Phyllanthus gaudichaudii* Muell.-Arg. var *marianus* (Müll. Arg.) Müll. Arg., in DC. Prodr. 15(2): 300. 1866. **TYPE. MARIANA ISLANDS: Guam:** C. Gaudichaud-Beaupré 139(holotype: G-DC).

**Distribution.** Endemic to the Mariana Islands (Guam) where it occurs on limestone and basaltic soils in old fields and grasslands and disturbed native and secondary vegetation at ca. 60–150 m elevation.

**Note.** The ovate leaves with short acuminate to rounded apices and glabrous columnar style 1.5–2 mm long exserted beyond the calyx lobes are characteristic of this species. Collections from Caroline Islands (Pohnpei) previously identified as this species are here considered to represent *Phyllanthus senyavinianus*.

**36.** *Phyllanthus marianus* **Müll. Arg.**, Linnaea 32:17. 1863.

*Diasperus marianus* (Müll. Arg.) Kuntze, Revis. Gen. Pl. 2: 600. 1891. **TYPE. MARIANA ISLANDS: Guam:** *s.l.*, 17 March to 3 April 1819, C. Gaudichaud-Beaupré s. n. (holotype: G-DC).

**Distribution.** Endemic to the Mariana Islands (Guam, Aguijan, Agrihan, Alamagan, Anathan, Asuncion, Guguan, Maug, Pagan, Rota, Saipan, Sarigan, Tinian) and the Caroline Islands (only on Ulithi Atoll). On Guam it is common on limestone cliffs and terraces.

**37.** *Phyllanthus melvilliorum* **(Airy Shaw) W.L. Wagner & Lorence, comb. nov.**

urn:lsid:ipni.org:names:77112715-1

**Basionym:** *Glochidion melvilliorum* Airy Shaw., Kew Bull. 25: 487. 1971. **TYPE. FUJI ISLANDS: Viti Levu:** Nausori Highlands, Nandronga and Navosa Province, 2 May 1962, R. & E.F. Melville & J.W. Parham 7048 (holotype: K; isotypes: BISH, SUVA).

**Distribution.** Endemic to Fiji and known only from the vicinity of the type locality at 600–670 m in dense or mixed forest (Smith 1981).

**38.** *Phyllanthus multilobus* **(A.C. Sm.) W.L. Wagner & Lorence, comb. nov.**

urn:lsid:ipni.org:names:77112716-1

**Basionym:** *Glochidion multilobum* A.C. Sm., Fl. Vitiensis Nova 2: 484, 493. 1981. **TYPE. FIJI ISLANDS: Vanua Levu:** Thakaundrove Prov., SW slope of Mt. Batini, 28 Nov 1933, A.C. Smith 606 (holotype: BISH-142931; isotypes: many indicated, but not specific as to where deposited).

**Distribution.** Endemic to Fiji and know only from Mt. Batini and Mt. Seatura on Vanua Levu from 300 to 800 m in dense or crest forest (Smith 1981).

**39.** *Phyllanthus nadeaudii* **(J. Florence) W. L. Wagner & Lorence, comb. nov.**

urn:lsid:ipni.org:names:77112717-1

**Basionym:** *Glochidion nadeaudii* J. Florence, Bull. Mus. Natl. Hist. Nat., B, Adansonia 18: 253. 1996. **TYPE. SOCIETY ISLANDS: Moorea:** Pao Pao, crête N du Mt. Fairurani. 670 m, 149°47’ W, 17°30’ S, 14 May 1987, J. Florence 8287 (holotype: P; isotypes: BISH!, PAP, US!).

**Distribution.** Endemic to the Society Island of Moorea at 420–800 m, where it typically occurs on high ridge slopes and crests with *Weinmannia*, *Metrosideros*, *Dicranopteris*, and *Nephrolepis* (Florence 1997).

**40.** *Phyllanthus orohenense* **(J. W. Moore) W. L. Wagner & Lorence, comb. nov.**

urn:lsid:ipni.org:names:77112718-1

**Basionym:** *Glochidion orohenense* J. W. Moore, Bernice P. Bishop Mus. 16: 6. 1940. **TYPE: SOCIETY ISLANDS: Tahiti:** south side of Mt. Orohena, 1300 m, 14 May 1927, L. H. McDaniels 1312(holotype: BH; isotype: BISH!).

**Distribution.** Endemic to the Society Island of Tahiti where it is rare and localized at 900–1750 m, in cloud forest with *Metrosideros*, *Weinmannia*, and *Alsophila* (Florence 1997).

**41.** *Phyllanthus otobedii* **W. L. Wagner & Lorence, nom. nov.**

urn:lsid:ipni.org:names:77112719-1

**Replaced name:** *Glochidion palauense* Hosokawa, Trans. Nat. Hist. Soc. Taiwan 25: 22. 1935. **TYPE. CAROLINE ISLANDS: Palau:** Aurapushekaru I. (Oropusyakaru-to), S. of Koror I., 8 Oct 1933, T. Hosokawa 7453 (holotype: TAI; isotype: BISH!, MICH, US!, Z).

**Distribution.** Endemic to Palau on Babeldaob, Anguar, Ngerechong, Ulebsechel, Ulong, and the Rock Islands (Koror, Ngerukeuid, Ngeruktabel). On Babeldaob this species occurs on basaltic soils in evergreen wet forest and savannas at about 100 m elevation. On the Rock Islands it occurs on limestone substrate near sea level (2–5 m elevation) in evergreen coastal forest and cliff vegetation. This glabrous species is distinguished by its female flowers with a conical-columnar pistil 3 mm long with a cylindrical style and shortly 6–7-lobed stigma.

**Etymology.** We are pleased to name this species for Mr. Demei O. Otobed, president of the board of directors of the Belau National Museum, who has done so much to advance the study and conservation of Palau’s biodiversity.

**Note.** Some Palau collections of this species were identified as *Glochidion macrosepalum*.

**42.** *Phyllanthus pacificus* **Muell. Arg,** Linnaea 32 : 31. 1863.

*Diasperus pacificus* (Müll. Arg.) Kuntze, Revis. Gen. Pl. 2: 600. 1891. **TYPE. MARQUESAS ISLANDS: Nuku Hiva:** D.E.S.A. Jardin 122 (lectotype: P, designated by Florence, Fl. Polynésie Française 1: 123. 1997; isolectotype: G-DC [2], P).

*Phyllanthus pacificus* Müll. Arg. var. *uapensis* F. Br., Bernice P. Bishop Mus. Bull. 130 : 138. 1935, syn. nov. **TYPE. MARQUESAS ISLANDS: Ua Pou:** 9 Sep 1922, E. H. Quayle s.n. (holotype: BISH-143921! & 508277!; isotype: BISH).

*Phyllanthus pacificus* Müll. Arg. var. *quaylei* F. Br., Bernice P. Bishop Mus. Bull. 130: 139. 1935, syn. nov. **TYPE. MARQUESAS ISLANDS: Nuku Hiva:** s*.l.*, 15? Oct 1922, E. H. Quayle 1341 (holotype: BISH-508703!).

*Phyllanthus pacificus* Müll. Arg. var. *uahukensis* F. Br., Bernice P. Bishop Mus. Bull. 130: 139. 1935, syn. nov. **TYPE: MARQUESAS ISLANDS: Hiva Oa:** Kopaafaa, 2770 ft, 2 Aug 1929, E.P. Mumford & A.M. Adamson 488 (lectotype: BISH-508705!; designated by St. John, Phytologia 33: 420. 1976). **Additional syntypes: MARQUESAS ISLANDS: Ua Huka:** *s.l.*, 9 Nov 1922, E. H. Quayle 1781 (BISH-508704! & -143920!).

**Distribution.** Endemic to the Marquesas Islands (Nuku Hiva, Hiva Oa, Tahuata, Ua Pou, Ua Huka, and Fatu Hiva) where it is widespread and occurs from about 25 to 1085 m, growing in open areas or on rocky slopes and cliffs in secondary vegetation with *Dicranopteris* and grasses. At higher elevations it grows among fern cover in *Metrosideros*-*Weinmannia* wet shrubland and forest (Florence 1997).

**43.** *Phyllanthus palauensis* **Hosokawa**, Trans. Nat. Hist. Soc. Taiwan 25: 19. 1935. **TYPE. CAROLINE ISLANDS: Palau:** Babeldaob Island, near Almatin at low altitudes, 17 Sep 1933, T. Hosokawa 6921 (holotype: TAI; isotype: GH).

**Distribution.** Endemic to Palau (Babeldaob, Ngemelachel, Ulong) and Rock Islands (Koror, Mecherchar, Ngerukeuid, Ngerekebesang, Ngeruktabel) where it occurs to at least 30 m elevation on volcanic soils in wet forests along streams and in savannah vegetation.

**44.** *Phyllanthus papenooense* **(J. Florence) W. L. Wagner & Lorence, comb. nov.**

urn:lsid:ipni.org:names:77112720-1

**Basionym.** *Glochidion papenooense* J. Florence, Bull. Mus. Natl. Hist. Nat., B, Adansonia 18: 254. 1996. **TYPE: SOCIETY ISLANDS: Tahiti:** Papenoo, Ofetanu, 160 m, 149°26’ W, 17°38’ S, 9 Sep 1989, J. Florence 9901 (holotype: P; isotypes: BISH!, PAP).

**Distribution.** Endemic to the Society Island of Tahiti where it is apparently rare and known only from Papenoo Valley up to about 650 m in riparian forest with *Hibiscus* and *Neonauclea* invaded by *Miconia calvescens* DC. (Florence 1997).

**45.** *Phyllanthus pergracilis* **Gillespie**, Bernice P. Bishop Mus. Bull. 91: 18. 1932. **TYPE. FIJI ISLANDS: Viti Levu:** Naitasiri Province, Tamavua woods, 11 km from Suva, 150 m, 9 Aug 1927, J.W. Gillespie 2122 (holotype: BISH-508710!; isotypes: GH, UC).

**Distribution.** Endemic to Fiji and known only from Viti Levu at 30–1200 m (Smith 1981; Webster 1986).

**46.** *Phyllanthus pinaiensis* **S.L.Welsh**, Flora Societensis 112. 1998.

**Basionym:** *Phyllanthus urceolatus* Baill., Adansonia 2: 239. 1862. *Diasperus urceolatus* (Baill.) Kuntze, Revis. Gen. Pl. 2: 601. 1891, non *Phyllanthus urceolatus* Noronha (1790). **TYPE. SOC IETY ISLANDS: Tahiti: “**Nouvelle-Caledonie, Port de France”, E. Vieillard 336 (holotype: P; isotype: P).

**Distribution.** Endemic to the Society Islands of Moorea, Raiatea, and Tahiti from 150–830 m, usually in understory of valley forest with *Hernandia*, *Hibiscus*, and *Neonauclea* or sometimes on ridge crests. Not collected on Tahiti since the end of the 19th century but apparently still frequent on Moorea (Florence 1997).

**Note.** According to Florence (1997) the type of *Phyllanthus urceolatus* Baill. is from Tahiti, not New Caledonia. This is likely due to a labeling error.

**47.** *Phyllanthus pitcairnense* **(H. St. John) W. L. Wagner & Lorence, comb. nov.**

urn:lsid:ipni.org:names:77112721-1

**Basionym.** *Glochidion pitcairnense* (F.Br.) H.St.John, Trans. Roy. Soc. New Zealand, Bot. 1: 187. 1962. *Glochidion tahitense* var. *pitcairnense* F. Br., Bernice P. Bishop Mus. Bull. 130: 142. 1935. **TYPE: PITCAIRN ISLANDS:** *s.l.*, 1922, E. H. Quayle s.n. (holotype: BISH-508246! & -508247!).

**Distribution.** Endemic to the Pitcairn Islands (Henderson and Pitcairn), from about 30 to 270 m elevation. On Henderson it occurs at 30 m on eroded calcarenite in beach forest and scrub with *Nesoluma*, *Pisonia*, and *Xylosma*. On Pitcairn it has been collected at 270 m in secondary upland vegetation with grasses and ferns.

**48.** *Phyllanthus podocarpus* **Müll. Arg.**, Flora 48: 388. 1865*.Diasperus podocarpus* (Müll. Arg.) Kuntze, Revis. Gen. Pl. 2: 600. 1891. *Glochidion podocarpum* (Müll. Arg.) C.B. Robinson, Philippine J. Sci. Bot. 6: 300. 1911. **TYPE. FIJI ISLANDS:** *s. l.*, 1840, U.S. Expl. Exped. s.n. (holotype: probably G; isotype: US!).

**Distribution.** Endemic to Fiji and know only from the type collection.

**49.** *Phyllanthus ponapense* **(Hosokawa) W. L. Wagner &** **Lorence, comb. nov.**

urn:lsid:ipni.org:names:77112722-1

**Basionym.** *Glochidion ponapense* Hosokawa, Trans. Nat. Hist. Soc. Taiwan 25: 24. 1935. **TYPE: CAROLINE ISLANDS: Pohnpei:** summit of Mt. Troton, 13 Aug 1933, T. Hosokawa 5770 (holotype: TAI).

*Glochidion excorticans* Fosberg var. *calvum* Fosberg, Willdenowia 20: 261. 1991, syn. nov. **TYPE: CAROLINE ISLANDS: Pohnpei:** 1913-1914, C. L. Ledermann 13333 (holotype: B-bc100249513!).

**Distribution.** Endemic to Pohnpei, this species occurs from lowland wet forest up into montane cloud forest on summits from 20 to 732 m elevation.

**Note.** This species is characterized by its female flowers with a short, glabrous, depressed-globose ovary 0.5 mm long and glabrous columnar style 1-1.5 mm long with a 6-7-dentate stigma. Some collections of *Phyllanthus ponapense* were previously identified as *Glochidion marianum* or *Glochidion ramiflorum*.

**50.** *Phyllanthus raiateaensis* **W. L. Wagner & Lorence, nom. nov.**

urn:lsid:ipni.org:names:77112723-1

**Replaced name:** *Glochidion moorei* P. T. Li, Acta Phytotax. Sin. 20: 117. 1982, non *Phyllanthus moorei* M. Schmid (1991). *Glochidion salicifolium* J. W. Moore, Bernice P. Bishop Mus. Bull. 226: 13. 1963, non *Glochidion salicifolium* (Baill.) Müll. Arg. (1863) nec *Phyllanthus salicifolius* Baill (1862). **TYPE. SOCIETY ISLANDS:** **Raiatea:** Temihani Plateau, 5 Oct 1934, H. St. John 17250 (holotype: BISH-508232!; isotype: BISH).

**Note.** The sheet (BISH-142814) was considered by Florence (1997) to not be part of the type because it was pubescent vs. glabrous as in the holotype. The full variation of *Phyllanthus raiateaensis* is not well understood, but Florence accepted both specimens as this species. Without further supporting information that this really represents a mixed collection it seems best to accept the second sheet as an isotype. However, even if the second sheet is not accepted as an isotype, the first sheet is clearly the holotype as it was explicitly stated as such and shown in a figure in the original publication.

**Distribution.** Endemic to the Society Island of Raiatea where it is known from the Temehani plateau region at 435–750 m, occurring in wet forest with *Metrosideros*, *Weinmannia*, and *Myrsine* and in open marshland with *Metrosideros* and species of Cyperaceae (Florence 1997).

**51.** *Phyllantus raivavense* **(F. Br.) W. L. Wagner & Lorence, comb. nov.**

urn:lsid:ipni.org:names:77112724-1

**Basionym.** *Glochidion raivavense* F. Br., Bernice P. Bishop Mus. Bull. 130: 142. 1935. **TYPE. AUSTRAL ISLANDS: Raivavae:** 23 Mar 1922, A. M. Stokes 43 (holotype: BISH-508248!).

**Distribution.** Endemic to the Austral Islands (Raivavae, Rurutu, and Tubuai). It occurs at 10–340 m in primary and secondary vegetation including riparian forest with *Aleurites*, *Hernandia*, *Hibiscus tiliaceus*, and *Metrosideros*, and on dry slopes or crests with *Celtis*, *Dicranopteris*, *Xylosma*, and grasses (Florence 1997).

**52.** *Phyllanthus rapaense* **(J. Florence) W. L. Wagner & Lorence, comb. nov.**

urn:lsid:ipni.org:names:77112725-1

**Basionym.** *Glochidion rapaense* J. Florence, Bull. Mus. Natl. Hist. Nat., B, Adansonia 18: 258. 1996. **TYPE. AUSTRAL ISLANDS: Rapa:** flanc SE du Mt. Pukumia, 150 m, 144°19’ W, 27°36’ S, 5 Feb 1984, J. Florence 6465 (holotype: P; isotypes: BISH!, K, PAP, US!).

**Distribution.** Endemic to the Austral Island of Rapa, at 50–330 m in mesic forest with *Metrosideros*, *Meryta*, and *Freycinetia*, and sometimes on wet rocks or cliffs (Florence 1997).

**53.** *Phyllanthus rupiinsularis* **Hosokawa,** Trans. Nat. Hist. Soc. Taiwan 25: 19. 1935. **TYPE. CAROLINE** **ISLANDS: Palau:** upon a coral islet near the island of Urktable [Ngeruktabel], 15 Oct 1933, T. Hosokawa 7534 (holotype: TAI; isotype: GH, US [2]!).

**Distribution.** Endemic to Palau where it occurs on the Rock Islands (Ngerukeuid, Ngeruktabel, Ulong), on limestone substrate in coastal cliff vegetation near sea level to about 5 m elevation.

**54.** *Phyllanthus saffordii* **Merr.**, Philipp. J. Sci., C 9: 104. 1914. **TYPE. MARIANA ISLANDS:** Guam: hills back of Piti, 100 m, Oct 1911, R. C. McGregor 476 (lectotype: US- 01860446!, here designated).The type in PNH was destroyed during World War II.

**Distribution.** Endemic to the Maraiana Islands (Guam, Alamagan, Anatahan, Pagan, Saipan, and Tinian). This species occurs in savannah vegetation.

**55.** *Phyllanthus st-johnii* **W. L. Wagner & Lorence, nom. nov.**

urn:lsid:ipni.org:names:77112726-1

**Replaced name:** *Glochidion myrtifolium* J. W. Moore, Bernice P. Bishop Mus. Bull. 226: 10. 1963. **TYPE. SOCIETY ISLANDS: Raiatea**: S ridge of Ereeo Valley, 9 Oct 1934, H. St. John 17328 (holotype: BISH-508233!; isotype: BISH).

*Glochidion longipedicellatum* J.W.Moore, Bernice P. Bishop Mus. Bull. 226: 9. 1963, nom. illeg., non Yamamoto (1933). *Glochidion longipes* P.T.Li, Acta Phytotax. Sin. 20:117. 1982. **TYPE. SOCIETY ISLANDS: Raiatea**: south side of Toahiva Valley, 200 m, 7 Oct 1934, H. St. John 17305 (holotype: BISH-508155!; isotype: BISH).

**Distribution.** Endemic to the Society Islands (Bora Bora, Moorea, Raiatea, and Tahaa) where it occurs at 30–680 m in primary or secondary mesic or wet forest with *Hibiscus tiliaceus* and *Nauclea* in valleys, or with *Dicranopteris*, *Metrosideros*, and *Psidium* on slopes and ridges (Florence 1997).

**56.** *Phyllanthus samoanus* **(Müll. Arg.) W. L. Wagner & Lorence, comb. et stat. nov.**

urn:lsid:ipni.org:names:77112727-1

**Basionym:** *Phyllanthus ramiflorus* (J.R. Forst. & G. Forst.) Müll. Arg. var. *samoanus* Müll. Arg. in A.P.de Candolle, Prodr. 15(2): 289. 1866. *Glochidion ramiflorum* J.R. Forst. & G. Forst. var. *samoanum* (Müll. Arg.) Pax, Bot. Jahrb. Syst. 25: 645. 1898. **TYPE. SAMOAN ISLANDS:** *s.l.*, U.S. Expl. Exped. s.n. (holotype: probably G-DC).

*Phyllanthus gaudichaudii* var. *samoanus* Müll. Arg. in A.P.de Candolle, Prodr. 15(2):300. 1866. *Glochidion cuspidatum* var. *samoanum* .(Müll. Arg.) Pax, Bot. Jahrb. Syst. 25:645. 1898. **TYPE. SAMOAN ISLANDS:** *s.l.*, U.S. Expl. Exped. s.n. (holotype: probably G-DC).

**Distribution.** Endemic to the Samoan Islands (Savai`i, Upolu, Tutuila, Aunu`u, Ofu, Olosega, and Ta`u) at 60–1000 m disturbed forest, secondary forest, and pastures (Whistler 1980).

**Note.** Even though the varietal name was published under an illegitimate species it is legitimate under Art. 55.2 of the ICBN and is available for use at the specific level. These Samoan collections were previously referred to *Glochidion ramiflorum*, butwere considered by Smith (1981) to be a separate species. He considered *Glochidion ramiflorum* to be a species from New Guinea to New Hebrides [see Excluded Names].

**57.** *Phyllanthus seemannii* **(Müll. Arg.) Müll. Arg.**, Flora 48: 374. 1865 (as *seammanianus*).

**Basionym.** *Glochidion seemannii* Müll. Arg., Linnaea 32: 63. 1863 (as *seemanni*). *Diasperus seemannii* (Müll. Arg.) Kuntze, Revis. Gen. Pl. 2: 600. 1891. **TYPE. FIJI ISLANDS:** Kadavu: *s.l.*, 1860, B.C. Seemann 413 (holotype: probably G; isotypes: BM, K).

*Phyllanthus venulosus* Müll. Arg., J., Flora 48:374. 1865. *Diasperus venulosus* (Müll. Arg.) Kuntze, Revis. Gen. Pl. 2: 601. 1891. *Glochidion venulosum* (Müll. Arg.) P.T.Li, Guihaia 14: 131. 1994. **TYPE. FIJI ISLANDS:** *s.l.*, 1840, *U.S. Expl. Exped. s.n.* (Holotype: probably G; Isotypes: GH, US).

**Distribution.** Endemic to Fiji where it is known from Viti Levu, Ovalau, Vanua Levu, Taveuni, and Moala, but Smith (1981) thought it is most likely more widespread, at 0–1150 m, in dense dry or secondary forest or on more open hillsides.

**58.** *Phyllanthus senyavinianus* **(Glassman)** **W. L. Wagner & Lorence, comb. nov.**

urn:lsid:ipni.org:names:77112728-1

**Basionym.** *Glochidion senyavinianum* Glassman, Bernice P. Bishop Mus. Bull. 209: 71. 1952. **TYPE. CAROLINE ISLANDS: Pohnpei:** Mt. Ninani, 731 m, 17 Aug 1949, S. Glassman 2884 (holotype: US-02158415!; isotypes: BISH, OKL).

*Glochidion puberulum* Hosokawa, Trans. Nat. Hist. Soc. Taiwan 25:23. 1935, non *Phyllanthus puberulus* Miq. ex Baill., syn. nov. (1858). **TYPE. CAROLINE ISLANDS: Pohnpei:** 8 Aug 1933, T. Hosokawa 5523 (holotype: TAI; isotype: US!).

*Glochidion excorticans* Fosberg, Willdenowia 20: 260. 1991, syn. nov. **TYPE. CAROLINE ISLANDS: Pohnpei:** 1913-1914, C. L. Ledermann 13643x (holotype: B!).

**Distribution.** Caroline Islands, known from Pohnpei and Chuuk (Fano, Dublon, Nomwin, Melot Moen, Romonum, Tol, Udot, and Uman). On Pohnpei it occurs from near sea level (2 m) to 770 m in primary and secondary lowland and montane wet forest and summit cloud forest. On Moen it occurs in lowland areas among mangrove swamps near sea level and on slopes and ridges where said to be common in agroforest and secondary forest. Habitat is unknown on the other islands of Chuuk.

**Note.** This species is characterized by its variably pubescent stems and densely hirtellous pistil and capsules. Collections from Chuuk resemble *Phyllanthus senyavinianus* in having a densely hirtellous ovary and style, but the pistil is comparatively shorter and only as long as the calyx lobes, and the leaves are narrowly ovate-oblong. These collections from Chuuk were previously identified as *Glochidion puberulum* and are here tentatively included under *Phyllanthus senyavinianus*, but may represent an undescribed species. Chuuk collections of *Phyllanthus kanehirae* differ in having female flowers with a glabrous pistil nearly twice as long as the calyx lobes. Some of these collections were previously identified as *Glochidion puberulum.* Certain Pohnpei collections of *Phyllanthus senyavinianus* were previously identified as *Glochidion ramiflorum* or *Glochidion marianum*.

**59.** *Phyllanthus smithianus* **G. L. Webster**, Pacific Sci. 40: 99. 1986 (1987). **TYPE. FIJI ISLANDS: Viti Levu:** Rewa, woods at summit of Mt. Korombamba, 381 to 427 m, 09 Jul 1968, G.L. Webster, R. Hildreth & I. Kuruvoli 14078 (holotype: DAV; isotypes: BI, SH, GH, NY, US!).

**Distribution.** Endemic to Fiji on the southern part of Viti Levu at 50–430 m (Smith 1981; Webster 1986).

**Note.** This distinctive species was treated as *Phyllanthus* sp. by Smith (1981, p.464).

**60.** *Phyllanthus societatis* **Müll. Arg.**, in DC. Prodr. 15(2): 364. 1866. *Diasperus societatis* (Müll. Arg.) Kuntze, Revis. Gen. Pl. 2: 601. 1891. **TYPE. SOCIETY ISLANDS:** *s. l.*,1838–1842, U.S. Expl. Exped. s.n. (holotype: G-DC; isotype: US!).

**Distribution.** Known from the central southern Pacific region, ranging from Nauru to the Tuamotu Islands (Makatea) and Cook Islands (Aitutaki, Atiu, Mauke, and Mitiaro). Restricted to lowland calcareous substrates, usually in clearings or sunny sites in forest with *Guettarda*, *Hibiscus*, *Homalium*, and *Pandanus* (Florence 1997).

**61.** *Phyllanthus taitensis* **(Baill. ex Müll. Arg.) Müll. Arg.**, Flora 48: 380. 1865.

**Basionym.** *Glochidion taitense* Baill. ex Müll. Arg., Linnaea 32: 66. 1863. *Diasperus taitensis* (Baill. ex Müll. Arg.) Kuntze, Revis. Gen. Pl. 2: 601 (1891). **TYPE. SOCIETY ISLANDS: Tahiti:** 1847, J. Lépine 209(holotype: G-DC; isotypes: P [3]).

*Phyllanthus taitensis* (Baill. ex Müll. Arg.) Müll. Arg. var. *glabrescens* Müll. Arg. in A.P.de Candolle, Prodr. 15(2): 301. 1866. **TYPE. SOCIETY ISLANDS: Tahiti:** 1838–1842, U.S. Expl. Exped. s.n. (holotype: G-DC; isotype: US!).

*Glochidion ramiflorum* J.R. Forst. & G. Forst. var. *macrophyllum* Müll. Arg., Linnaea 32: 63. 1863. *Phyllanthus ramiflorus* (J.R. Forst. & G. Forst.) Müll. Arg.var. *macrophyllus* (Müll. Arg.) Müll. Arg., Flora 48: 374. 1865. **TYPE. SOCIETY ISLANDS: Tahiti:** J.A. Moerenhout s.n. (lectotype: G, designated by Florence, Fl. Polynésie Française 1: 123. 1997; isolectotype: P).

**Distribution.** Endemic to the Society Islands of Moorea and Tahiti where widespread and common from 50 to 1500 m, occurring from lowland wet forest with *Hibiscus tiliaceus* and *Neonauclea* in valleys to mid and high elevation wet forest with *Alstonia*, *Metrosideros*, *Streblus*, and *Weinmannia* (Florence 1997).

**62.** *Phyllanthus temehaniensis* **(J. W. Moore) W. L. Wagner & Lorence, comb. nov.**

urn:lsid:ipni.org:names:77112729-1

**Basionym.** *Glochidion temehaniense* J. W. Moore, Bernice P. Bishop Mus. Bull. 226: 15. 1935. **TYPE. SOCIETY ISLANDS: Raiatea:** Temehani Plateau, 600 m, 5 Oct 1934, H. St. John 17279 (holotype: BISH-508231!; isotype: P).

**Distribution.** Endemic to the Society Islands (Huahine, Raiatea, and Tahaa) where it occurs from 0 to 600 m in lowland vegetation such as coconut plantations and wet valleys with *Hibiscus* and *Neonauclea* to higher slopes and ridge crests with wet forest or shrubland of *Metrosideros*, *Weinmannia*, and Cyperaceae (Florence 1997).

**63.** *Phyllanthus tuamotuensis* **(J. Florence) W. L. Wagner & Lorence, comb. nov.**

urn:lsid:ipni.org:names:77112730-1

**Basionym.** *Glochidion tuamotuense* J. Florence, J.,  Fl. Polynésie Française 1: 98. 1997. **TYPE. TUAMOTU ISLANDS:** **Niau**: Maiahu, secteur oust, 146°20’ W, 16°11’ S, 2 m, 26 Mar 1990, J. Florence 10070 (Holotype: P; Isotypes: BISH!, CHR, DAV, K, L, P, PAP, PTBG!, US!).

**Distribution.** Endemic to the Gambier Islands (Taravai, Mangareva) and Tuamotu Islands (Niau), where it occurs from near sea level to 7–8 m on calcareous substrate. On Niau it grows in lowland forest with *Allophylus*, *Planchonella*, and *Xylosma*, whereas on Taravai it was collected in secondary vegetation with *Dicranopteris* and *Psidium* (Florence 1997).

**64.** *Phyllanthus virgatus* **G. Forst.**, Fl. Ins. Austral. Prodr. 65. 1786. *Phyllanthus simplex* var. *virgatus* (G.Forst.) Müll. Arg., Linnaea 32: 32. 1863, nom. illeg. *Diasperus virgatus* (G.Forst.) Kuntze, Revis. Gen. Pl. 2: 597. 1891. **TYPE. SOCIETY ISLANDS: Tahiti:** Not designated.

**Distribution.** Widespread, but increasingly rare on Pacific Islands from Vanuatu, Fiji, Samoa, Tonga, Austral, Society and Cook islands (Webster 1986), and Caroline Islands (Yap) and Mariana Islands (Guam) in Micronesia, from 15 to 500 m elevation.

**Note.** Smith (1981) treated *Phyllanthus virgatus* as native in Asia, but likely naturalized in the Pacific as did Florence (1997). Webster (1986) found a number of morphological features (smaller seeds, short fruiting pedicels, smooth to slightly roughened ovaries, and an irregularly dissected disk) that distinguish the Pacific island populations from the mainland Asian ones, and thus the Asia plants are most likely a different species, *Phyllanthus simplex* Retz. Considering these differences the Pacific island *Phyllanthus virgatus* is likely a native and not found outside of Pacific islands. A complex set of considerations would be involved to make a proper lectotypification for this species. A. C. Smith (1981, p. 464) suggested that one of two collections at BM (Banks & Solander s.n.) be selected by a specialist as the lectotype. Webster (1986) incorrectly considered Smith’s comments to be a lectotypification, and Nicolson and Fosberg (2004) summarized the Cook voyage materials available, including Foster collections. They thought it inappropriate to select the Banks and Solander collection as the lectotype while also pointing out that a specialist needs to make the selection because the original material could represent a mixture of this species or *Phyllanthus simplex* Retz. or *Phyllanthus maderaspatensis* L.

**65.** *Phyllanthus vitiensis* **Müll. Arg.**, Flora 48: 374. 1865. *Diasperus vitiensis* (Müll. Arg.) Kuntze, Revis. Gen. Pl. 2: 601. 1891. *Glochidion vitiense* (Müll. Arg.) Gillespie, Bernice P. Bishop Mus. Bull. 91: 17. 1932. **TYPE. FIJI ISLANDS:** *s. l.*, 1840, U.S. Expl. Exped. s.n. (holotype: probably G; isotype: US!).

*Glochidion concolor* var. *obovatum* Müll. Arg., Linnaea 32: 62. 1863. *Phyllanthus concolor* var. *obovatus* (Müll. Arg.) Müll. Arg. in A.P.de Candolle, Prodr. 15(2): 290. 1866. **TYPE. FIJI ISLANDS: Viti Levu:** 1860, B. C. Seemann 412 (holotype: probably G; isotypes: BM, K).

**Distribution.** Endemic to Fiji and known from Viti Levu, Kadavu, Nairrai, Moala, Kanacea, and Vanua Balavu, at 0–590 m in dense dry forests, open rolling hills, and grassy slopes.

**66.** *Phyllanthus vitilevuensis* **W.L. Wagner & Lorence, nom. nov.**

urn:lsid:ipni.org:names:77112731-1

**Replaced name.** *Glochidion collinum* A.C. Sm., Fl. Vitiensis Nova 2: 486, 494. 1981, non *Phyllanthus collinus* Domin (1928). **TYPE.** **FIJI ISLANDS: Viti Levu**: Naitasiri, N portion of Rairaimatuku Plateau, between Mt. Tomanivi (Mt. Victoria) and Nasonggo, 870 to 970 m, 18 Sep 1947, A.C. Smith 6148 (holotype: BISH-142913; isotype: US!).

**Distribution.** Endemic to Fiji and known only from the interior of Viti Levu at 850–1150 m in dense forest (Smith 1981).

**67.** *Phyllanthus websteri* **(Fosberg) W. L. Wagner & Lorence, comb. nov.**

urn:lsid:ipni.org:names:77112732-1

**Basionym.** *Glochidion websteri* Fosberg, Willdenowia 20: 262. 1991. **TYPE. CAROLINE ISLANDS: Palau:** 1914, C. L. Ledermann 14507 (holotype: B-bc100241068!).

**Distribution.** Caroline Islands, Pohnpei, known only from the type without specific locality.

**Note.** A glabrous species to date know only from the type collection which has only pistillate flowers. The glabrous pistil is cylindrical with a columnar style 2-2.5 mm long exserted well beyond the calyx lobes. It most closely resembles and may be conspecific with *Phyllanthus ponapense*. This entity should be carefully evaluated in the context of an overall review of *Phyllanthus* in Micronesia.

**68.** *Phyllanthus wilderi* **(J. Florence) W. L. Wagner & Lorence, comb. nov.**

urn:lsid:ipni.org:names:77112733-1

**Basionym.** *Glochidion wilderi* J. Florence, Fl. Polynésie Française 1: 99. 1997. **TYPE. TUAMOTU ISLANDS: Makatea:** Vaitepaua Ouest, piste de Temao, 45 m, 148°16’ W, 15°49’ S, 31 Jan 1988, J. Florence 9073 (holotype: P; isotypes: BISH!, CHR, DAV, K, P, PAP, US!).

**Distribution.** Endemic to the Gambier Islands (Mangareva) and Tuamotu Islands (Makatea). On Makatea it occurs from 45–75 m on calcareous substrate in primary forest with *Guettarda* and *Pandanus* or in degraded *Homalium* forest. On Mangareva it was collected in relict primary or secondary forest and cliffs up to 350 m elevation (Florence 1997).

**69.** *Phyllanthus wilkesianus* **Müll. Arg.**, in DC., Prodr. 15(2): 396. 1866. *Diasperus wilkesianus* (Müll. Arg.) Kuntze, Revis. Gen. Pl. 2: 601. 1891. **TYPE. FIJI ISLANDS:** *s. l.*, 610 m, 1840, U.S. Expl. Exped. s.n. (holotype: G, isotype: GH).

**Distribution.** Endemic to Fiji and known only from two localities: Nadarivatu, Viti Levu and Macuata Range, Vanua Levu at 100–800 m (Smith 1981).

### Naturalized species

**70.** *Phyllanthus amarus* **Schumach. & Thonn**., Kongel. Danske Vidensk. Selsk. Skr., Naturvidensk. Math. Afd. 4: 195. 1829.

**Distribution.** Presumably native to the Neotropics, but now naturalized across tropical regions of the world; in the Pacific on Fiji, Austral,Cook, Gambier, Marquesas, Samoa, Society, Tuamotu Islands, and widespread in Micronesia (Carolines, Gilberts, Marshalls, Marianas, Nauru, and Wake) at 0–600 m elevation.

**71.** *Phyllanthus debilis* **Klein ex Willd.**, Sp. Pl. 4: 582. 1805. *Phyllanthus niruri* var. *debilis* (Klein ex Willd.) Müll. Arg. in A.P.de Candolle, Prodr. 15(2): 407. 1866. *Diasperus debilis* (Klein ex Willd.) Kuntze, Revis. Gen. Pl. 2: 601. 1891.

**Distribution.** Native to India and Sri Lanka, but now widely naturalized; in the Pacific naturalized on Fiji, Society Islands (only Tahiti), Hawaiian Islands, and Micronesia (Caroline Islands, Mariana Islands, and Marshall Islands) at 0–450 m. Specimens from Palau referred to *Phyllanthus boninsimae* Nakai are *Phyllanthus debilis*.

**72.** *Phyllanthus tenellus* **Roxb.**, Fl. Ind., ed. 1832, 3: 668. 1832. *Diasperus tenellus* (Roxb.) Kuntze, Revis. Gen. Pl. 2: 601. 1891.

**Distribution.** Origin uncertain, but now naturalized across the Indo-Pacific region and currently known from the Hawaiian Islands (Kaua`i, O`ahu, Lana`i, Maui, Hawai`i), Austral Islands (Raivavae, Rurutu, and Tubaui), Gambier (Mangareva, and Taravai), Marquesas Islands (Nuku Hiva), Society Islands (Huahine, Moorea, Raiatea, Tahiti, and Tetiaroa), and in Micronesia the Caroline Islands (Pohnpei).

**73.** *Phyllanthus urinaria* **L.**, Sp. Pl.: 982. 1753.*Diasperus urinaria* (L.) Kuntze, Revis. Gen. Pl. 2: 601. 1891.

**Distribution.** Native to southern Asia and now naturalized pantropically; it is naturalized across the Pacific and currently known from Fiji, Austral Islands (Rurutu), Marquesas Islands (Fatu Hiva), Society Islands (Huahine, Moorea, Raiatea, Tahaa, and Tahiti), Caroline Islands and Maraiana (Guam) Islands in Micronesia, at 0–1200 m.

### Excluded names

*Glochidion ramiflorum* J.R. Forst. & G. Forst., Char. Gen. Pl.: 57. 1775. *Bradleia glochidion* Gaertn., Fruct. Sem. Pl. 2: 128. 1791, nom. illeg. *Phyllanthus ramiflorus* (J.R. Forst. & G. Forst.) Müll. Arg., Flora 48: 374. 1865, nom. illeg., non *Phyllanthus ramiflorus* (Aiton) Pers., Syn. Pl. 2: 591. 1807 [= *Flueggea suffruticosa* (Pall.) Baill., Étude Euphorb.: 502. 1858]. *Diasperus ramiflorus* (J.R.Forst. & G.Forst.) Kuntze, Revis. Gen. Pl. 2: 600. 1891. **TYPE. TANNA AND AMSTERDAM ISLANDS:** *s. l.*, J.R. & G. Forster s.n. (lectotype: BM, designated by Smith, Fl. Vitiensis Nova 2: 473. 1981).

We follow Smith (1981) in considering the material previously assigned to this name from Fiji and Samoa to represent other species (*Phyllanthus concolor* and *Phyllanthus samoanus*). We also exclude the use of the name for collections from Micronesia, and include them instead under *Phyllanthus cleistanthoides* (the majority)*, P. ponapense*, and *Phyllanthus senyavinianus*. There is currently no available name within *Phyllanthus* for the *Glochidion ramiflorum* because it would need a new name as there are two different names *Phyllanthus ramiflorus*, both illegitimate. We provide the nomenclature for *Flueggea suffruticosa* below to bring together the complex set of names involving the epithet *ramiflorus*. Given the uncertainty surrounding the delimitation of this species we refrain from providing a new name here.

*Flueggea suffruticosa* (Pall.) Baill., Étude Euphorb. 502. 1858.

*Xylophylla ramiflora* Aiton, Hort. Kew. 1: 376, nom. illeg. 1789[based on *Pharnaceum suffruticosum* Pall.]. *Phyllanthus ramiflorus* (Aiton) Persoon, Syn. Pl. 2: 591. 1807, nom. illeg. *Securinega ramiflora* (Aiton) Müll. Arg. in A.P.de Candolle, Prodr. 15(2): 449. 1866, nom. illeg. *Acidoton ramiflorus* (Aiton) Kuntze, Revis. Gen. Pl. 2: 592. 1891, nom. illeg.
